# “…still waiting for chloroquine”: the challenge of communicating changes in first-line treatment policy for uncomplicated malaria in a remote Kenyan district

**DOI:** 10.1186/1475-2875-13-258

**Published:** 2014-07-08

**Authors:** Vincent Okungu, Lucy Gilson

**Affiliations:** 1 KEMRI-Wellcome Trust Research Programme, P.O. Box 230, Kilifi, Kenya; 2 Health Economics Unit, University of Cape Town, Anzio Road, 7925, Observatory, Cape Town, South Africa; 3 Health Policy and Systems Division, University of Cape Town, Anzio Road, 7925, Observatory, Cape Town, South Africa; 4 London School of Hygiene and Tropical Medicine, London, UK

**Keywords:** Policy communication, Artemether-lumefantrine, Uncomplicated malaria

## Abstract

**Background:**

Widespread parasite resistance to first-line treatment for uncomplicated malaria leads to introduction of new drug interventions. Introducing such interventions is complex and sensitive because of stakeholder interests and public resistance. To enhance take up of such interventions, health policy communication strategies need to deliver accurate and accessible information to empower communities with necessary information and address problems of cultural acceptance of new interventions.

**Objectives:**

To explore community understanding of policy changes in first-line treatment for uncomplicated malaria in Kenya; to evaluate the potential role of policy communication in influencing responses to changes in first-line treatment policy.

**Methods:**

Data collection involved qualitative strategies in a remote district in the Kenyan Coast: in-depth interviews (n = 29), focus group discussions (n = 14), informal conversations (n = 11) and patient narratives (n = 8). Constant comparative method was used in the analysis. Being malaria-prone and remotely located, the district offered an ideal area to investigate whether or not and how policy communication about a matter as critical as change of treatment policy reaches vulnerable populations.

**Results:**

Three years after initial implementation (2009), there was limited knowledge or understanding regarding change of first-line treatment from sulphadoxine-pyrimethamine (SP) to artemether-lumefantrine (AL) for treatment of uncomplicated malaria in the study district. The print and electronic media used to create awareness about the drug change appeared to have had little impact. Although respondents were aware of the existence of AL, the drug was known neither by name nor as the official first-line treatment. Depending on individuals or groups, AL was largely viewed negatively. The weaknesses in communication strategy surrounding the change to AL included poor choice of communication tools, confusing advertisements of other drugs and conflicts between patients and providers.

**Conclusion:**

Effective health policy communication is important for the uptake of new drug interventions and adherence to treatment regimens. Besides, prompt access to effective treatment may not be achieved if beneficiaries are not adequately informed about treatment policy changes. Future changes in treatment policy should ensure that the communication strategy is designed to pass sustained, accurate and effective messages that account for local contexts.

## Background

Changes in first-line treatment for uncomplicated malaria have occurred from chloroquine to antifolate combination drugs and to the current artemisinin-based combination therapy (ACT). However, there is no uniformity across countries in the stages of change from one drug to another because there are differences in geographical distribution of resistance to any single antimalarial. Chloroquine (CQ) was the most widely used anti-malarial drug since it was first synthesized in 1934 and for many decades was the drug of choice for treatment of non-severe or uncomplicated malaria, until drug resistance greatly reduced its usefulness [1]. Among the antifolate combination drugs, sulphadoxine-pyrimethamine (SP), sulphalene-pyrimethamine (Metakelfin®) and sulphamethoxazole-trimethoprim (co-trimoxazole) were the most widely used in sub-Saharan Africa [1]. Antifolate combination drugs did not take long before widespread global resistance was noted and the WHO recommended their replacement with ACT for treatment of both severe and non-severe malaria. The artemisinin-based combinations have faster parasite clearance and fever resolution than the other drugs [1].

Changing treatment policy is a standard scientific procedure but such a change needs to be understood by the entire citizenry who are the targeted beneficiaries. To reach everyone with adequate and accurate information about the change in treatment policy, there is a need for concerted health communication. As noted by [2] poor health communication is a major contributor to misuse of health care, non-adherence to treatment regimen and rising cost of health care. Health communication refers to passing information about health care that is understood by the target population [3] so as to allow take-up of an intervention. In disseminating information about the change in first-line treatment for uncomplicated malaria (i.e. from SP to artemether-lumefantrine), the target population was expected to understand and adjust their treatment expectations. Changing and implementing treatment policies are complex and fraught with challenges at every level. According to a report by [4] the challenges of changing and implementing treatment policies range from stakeholder interests, local and international bureaucracy to the potential resistance from the intended beneficiaries. For these challenges, [5,6] recommend accurate and effective communication of the treatment policy change to empower the public with information on the value of new interventions and facilitate uptake and proper utilisation. There has previously been little detailed study of what policy communication strategies have accompanied such drug regimen changes and implementation in most of the world. Neither has there been a detailed exploration of people’s perceptions about the changes in first-line treatment policy and how such perceptions influence uptake of health interventions.

### Context and overview of the treatment policy change in Kenya

Sulphadoxine-pyrimethamine (SP) was introduced in Kenya in 1998 but by 2001 the treatment failure rate was over 25%, a threshold for changing first-line treatment for uncomplicated malaria. The Kenya Drug Policy Technical Working Group met in 2003 and proposed artemether-lumefantrine (AL) (Coartem®) to replace SP because it was the only co-formulated ACT recommended by the WHO as having passed rigorous international regulatory scrutiny. Artemether-lumefantrine was finally rolled out country-wide in 2006 after much debate about sustainable financing and procurement regulations [7].

To implement the change, the MOH conducted training for health workers at the national, provincial and district levels. The training recommended parasitic diagnosis before administering Coartem® even though the government of Kenya [8] indicated that only 34% and 7.0% of peripheral facilities countrywide had microscopy and rapid diagnostic test kits respectively. In addition, multimedia approaches including road shows, radio, posters and newspapers were used to communicate the policy change directly to the public in a campaign that lasted three months [9].

## Methods

### Study setting

The study was conducted in a remote rural district on the Kenyan coast. The district was chosen because of the need to explore how policies get communicated and implemented in remote areas. More specifically, was the need to understand how policy changes in first-line treatment of uncomplicated malaria are implemented and how the same are understood by populations in hard-to-reach locations. The focus on a hard-to-reach population in an economically marginalized district was important to the study as it sought to understand how health interventions respond to the needs, characteristics and local circumstances of populations in remote locations. As stated by [2] vulnerable populations are most affected by lack of health information.

### Study design and data collection methods

A cross-sectional qualitative study was carried out in three purposively sampled administrative zones out of the four that make up the district. Two of the divisions were farthest from the urban Kinango district headquarters and the other was the location of the headquarters. This sampling approach was intended to capture any differences in experience between people living closest to the district headquarters and those from the farthest part of the district. Two villages per division were randomly selected to participate in the study.

Data were collected between January and February 2009 using document reviews, focus group discussions (FGDs) (n = 14), in-depth interviews (n = 29), informal conversations (n = 11) and patient narratives (n = 8). The number of interviews was not predetermined and interviews were stopped on saturation of information. Kiswahili was the main language of communication as it was the language of the respondents. Participants for FGDs were selected on the basis of age and gender. They included parents with young children aged below ten years because young children and pregnant women are most vulnerable to malaria infection. The range of topics explored in the FGDs included local information networks and sources of health information and perceptions about anti-malarials.

In-depth interviews were conducted with district health managers, community leaders and managers (in-charges) of six dispensaries. They provided information on the policy communication processes and their effectiveness, implementation challenges and uptake of AL. Table 1 summarizes the range of data collection methods applied in the study.

**Table 1:** Summary of data collection methods

**Tool**	**Number (n)**	**With whom?**	**Topics explored/Purpose**
1. In-depth interviews	29	District health managers (n = 3)	1. Malaria situation in the district
			2. Role in policy communication and implementation
			3. Uptake of AL in the district
			4. Effectiveness of communication strategies
		Managers (in-charges) at primary health facilities (n = 6)	1. Role in policy communication and implementation
			2. Perceived effectiveness of policy communication strategies
			3. Barriers and facilitators to policy changes/implementation at the local level
			4. Perceived community response to policy changes, attitudes to recommended drug
		Community leaders (n = 17) and mothers who had recently used a health facility (n = 3)	1. Experiences with malaria
			2. Types of health provision available
			3. Views on malaria drugs
			4. Views on recent changes in malaria drug
			5. Role in the change of drugs
			6. Sources of health information and preferred sources
			7. Usefulness of the information received
2. Focus Group Discussions (FGDs)	14	Young parents (male and female)	1. Views on recent malaria drug policy changes
			2. Communication about recent drug policy changes;
			3. What sort of communication, who got the communication, from what sources; usefulness of the communication, and what reactions to the communication.
			4. Influences over choice of health care
			5. Views over new drugs.
3. Informal conversations	11	Convenience sample from all age groups, both male and female	1. Experiences with various malaria drugs
			2. Perceptions about policy changes on 1^st^-line treatment for uncomplicated malaria
			3. Sources of information on government and facility matters
4. Patient narratives	8	Convenience sample of patients asked to talk about their experiences	Real life experiences when seeking treatment including experience with various drugs, the reactions to policy change and communication received about the new policies
5. Document reviews	6	Selected with the help of the hospital administrator	To enhance familiarity with content of policy documents and discussions, and to feedback on policy implementation process
6. Diary	1	Researchers’ experiences/observations	To enable constant reflection on the researchers’ experiences as well as helping in identifying emerging thoughts about issues and ideas that were followed up in subsequent interviews.

Informal conversations were based on chance and dwelt on communication about health issues and accounting for gender differences in the sources of health information as well as people’s perceptions about changes in malaria drugs. Patient or caretaker narratives were conducted with purposively selected patients or caretakers who were willing to talk to the researcher at the health facilities. Those selected were asked to tell their story about their illness or illness of the child; how it started, diagnosis, the range of treatment options undertaken and their effectiveness as well as their interaction with health workers. All interviews were audio-recorded except for informal conversations.

### Data analysis

All interviews were transcribed and translated into English. Coding and analysis were performed manually by developing a matrix for emerging categories and themes. Data from various sources were first analysed separately and emerging themes and categories compared later. The themes and categories centred on phrases, incidents and behaviour during interviews, discussions, and observations. In accordance with recommendations of [10,11], data from each theme or category were identified and analysed using the constant comparative method. Individual key informant interviews with health personnel and community members, for example, were separately compared and contrasted with each other. Such an approach, according to [12,13], helps in identifying patterns, consensus, differences, variations or contradictions, and in weighing the relative importance of information based on emphasis by the study participants. These informed the selection of data for presentation here.

The criterion for selecting data to present was based on relevance to the stated objectives. Data presented here were based on the level of importance as judged by emphasis and consistency in raising specific issues during interviews by study participants.

### Ethical approval

Ethical approval was sought from the Kenya Medical Research Institute (KEMRI) Ethical Review Committee and the University of Cape Town Ethical Review Board, Faculty of Health Sciences. All participants gave free and informed, written and/or verbal consent for the study.

## Results

Based on relevance to the objectives, the results were summarized and presented in relation to three sub-topics that represented the study participants’ main lines of understanding of AL and the changes in first-line treatment policy for uncomplicated malaria. The first part looks at the study population’s perceptions about general changes in first-line treatment policy for uncomplicated malaria over time, because such perceptions have a bearing on take-up of new drug interventions. The second part explores perceptions specific to the change from SP to AL including views of the study participants about the importance of AL as a first-line drug. The last part addresses barriers to health policy communication that most likely influenced poor uptake of AL and identifies key variables that could be useful for future interventions.

### Perceptions about why first-line drugs for uncomplicated malaria change

It was not clear from the literature or from the study participants how they got communication about earlier drug changes from chloroquine to SP. However, the majority of participants indicated that they learnt about new drugs mainly through media advertisements and at health facilities. Given the profit motivation of drug companies advertising through the media, most study participants strongly believed that any change in treatment policy was mainly driven by the desire for profit maximisation. This view was shared by all FGD participants, 17 key informant interviews, two district health managers, and four primary health facility in-charges. A community leader said of the changes:

“I think chloroquine is no longer available because the company associated with it had made a lot of money so the government thought it was time to give another company chance to make money. That explains why every time we have different types of drugs for malaria.”

Other study participants believed that drugs change because the government keeps testing different drugs to establish the most effectiveness one against malaria. Asked whether the change in malaria drugs was good for the community, most participants in 12 FGDs and nine informal interviews reported that it did not contribute to better treatment for malaria. They believed that any changes were confusing, as stated by female FGD participant:

“The change of drugs is very bad because when we are already used to one type of drug then one day we are given a new one whose name we do not know. This makes treatment difficult because we do not know which drug to use anymore….”

However, six key informants out of 20 at the village level understood the scientific basis for change of drugs and explained that drugs change due to treatment failure.

### Awareness of the change from SP to AL

Nearly all study participants were aware of the existence of AL but knew it only as “the drug with many tablets”. They had no idea that it replaced SP as the official first-line treatment for uncomplicated malaria. Only six people (four women and two men) among all study participants at the village level knew AL by name and recognized it as the official first-line anti-malarial. These individuals had formal jobs and better education than their peers had, and were residents of a village near the urban section of the study district.

There were mixed responses at the village level when asked about the history of changes in first-line treatment. Most respondents explained that drugs have changed from CQ to amodiaquine (AQ) or from SP to a number of painkillers as advertised through the radio. The respondents further explained that the variety of drugs mentioned reflected people’s preferences since there was no single drug that suited every malaria patient.

In the narratives, three malaria patients thought AL was a painkiller and most men in the more remote villages believed that malaria drugs were seasonal and preferred CQ to AL. A 51 year old man said in an FGD:

*“We do not know that drugs have changed, but we know there are seasons for malaria drugs and it is now the season for malaratab* (amodiaquine) *and that other one with many tablets. When chloroquine season returns, we will have no problems over which drug to use to treat malaria…”*

### Perceived efficacy of AL compared with other drugs

Whenever people have negative attitudes toward a health intervention, uptake is likely to be affected. A policy communication strategy that accompanies change of health interventions should pre-empt or change such negative attitudes. However, in the study area, a number of respondents regarded AL as harmful. This was identified in 14 in-depth interviews, nine FGDs and seven informal interviews. Older individuals (50 years and above) were particularly concerned that AL was more harmful and less effective than CQ. One male key informant remarked:

*“When taking the new drug* (Coartem), *we ask ourselves many questions: ‘Where are they from and why can’t we be given the ones that we know? What harm can they cause to us?’ This makes us afraid….. We use this drug but inwardly we ask ourselves why it is different from the ones we used before and whether they are any better in treating malaria….”*

The perceived harm in AL was further emphasized in the fact that it was not recommended for expectant mothers. A young man said:

“Recently, I asked my wife why she was given fewer drugs than those given to everyone who goes to the dispensary and she told me that the other drug with many tablets cannot be given to pregnant women. That confirms the new drug can be harmful to anyone else.”

Study participants classified malaria into two categories: ‘weak’ and ‘strong’. Weak malaria meant mild or non-serious illness in which the patient is not incapacitated in any way. Strong malaria on the hand meant that the patient is perceived to be seriously ill with malaria and requires urgent medical attention. A number of study participants complained that one type of drug cannot be used to treat both *strong* and *weak* malaria because using AL to treat ‘weak’ malaria when it is a strong drug could render it ineffective against ‘strong’ malaria and this could be fatal. Those who perceived AL as weak cited its numerous tablets (24 tablets for adult dose while SP had three tablets). A female FGD participant said of AL tablets:

“We heard that malaria drugs that we are used to are no longer good and hoped for a new and better drug, but that is not the case because a full dose of the new drug has 24 tablets instead of just a few. Why are the tablets so many if it is a good drug?”

According to health workers, some of those who thought that AL was weak were cases of recurrent malaria, which sometimes led to the judgement that AL was weak. A health worker confirmed that a few people who experienced recurrent malaria after taking AL were reluctant to use the drug again or seek any formal treatment at all. Those seeking formal treatment were keen to use a different health facility in the hope that they will not be given the same drug (AL). Should they be given the same drug, they were unlikely to take it, as confirmed by a female FGD participant:

“If we suffer from malaria soon after taking all those tablets, we demand for a different drug. That means we would go to a different dispensary but if that dispensary also issues the same drug, we bring those drugs home to keep, then turn to herbs or go to private clinics for injections....”

### Barriers to health policy communication during the change from SP to AL

A number of factors emerged as clearly influencing communication around the change of first-line drugs for uncomplicated malaria.

### Choice and preference of communication channels

**Figure 1:**
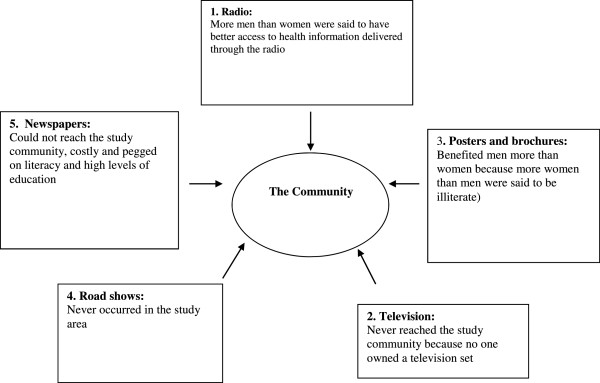
Communication channels used by the government to pass information about the change of first-line treatment policy to AL.

**Figure 2:**
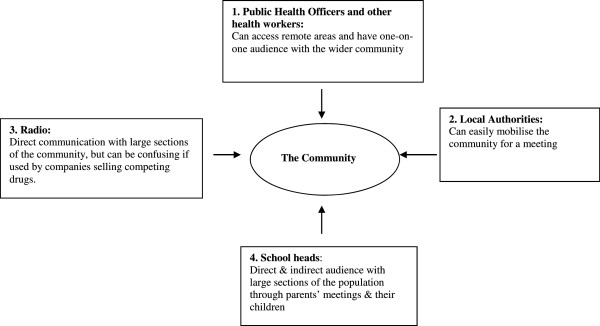
The study community’s preferred channels of communicating policy changes in first-line treatment for uncomplicated malaria and other health information in order of priority.

The findings indicate that the choice of policy communication strategies used by the government during the change to AL were not the most preferred in the study area. Figure 1 summarizes how information about the change from SP to AL was communicated to the public. These can be contrasted with Figure 2, which is a summary of the study community’s preferred communication channels for health policy communication.

Among the information, education and communication (IEC) tools used to facilitate the change from SP to AL, only radio and posters seemed to have reached the study district. However, the impact of these tools on the primary caretakers, mostly women, was negligible because most radios were owned and controlled by men. Although most people at health facilities were women and potentially had access to posters advertising the change to AL, widespread illiteracy made it impossible for them to read the posters. A young woman said of the posters:

“…all we see are graphics and pictures, which we think are decorations on the dispensary walls.”

Study participants emphasized that they preferred interpersonal communication to radios and posters and the best sources would be public health officers (PHOs), local authorities and teachers, and complemented by radio communication. The interpersonal communication allowed for exchange of views and prompt response to community concerns about the newly introduced drugs. For interpersonal communication however, the PHOs were not part of the change process for lack of resources to train them but a health manager acknowledged the mistake in leaving out the PHOs:

“It was a mistake to leave out the PHOs because their involvement would have ensured a more successful implementation of the change to AL.”

The health manager explained that emphasis on interpersonal communication would have best clarified that AL was the most suitable drug to treat malaria and could also have eliminated confusion over which drugs to use. A typical confusion over drugs was captured in the following statement:

“…the dispensary gave my wife what it said were the latest malaria drugs but the shopkeeper also had the latest drugs different from the dispensary. This is confusing.”

### Provider-patient conflicts

Conflicts were manifest in three areas: provider-patient communication, perceived health worker incompetence and professional integrity. Study participants complained of poor interpersonal relationships with health workers. In the narratives, two patients complained about rude health workers who refused to answer their queries about AL. A mother said:

“I wanted to know why I was given malaria drugs different from the ones I have known all along but the nurse asked me whether I came to be treated or to ask questions….”

Health workers admitted they rarely engaged patients about the change to AL because of the limited time to attend to large numbers of patients. At least one in-charge mentioned that it was not their responsibility to discuss issues of adherence with patients and saw no need to inform them about any changes in treatment:

“The patients are usually very many and we cannot engage each of them in lengthy conversations about adherence. Besides, I have never considered it important to engage the community on such issues as change of drugs....”

The perceived inability of health workers to give proper treatment affected use and adherence to AL. In six FGDs, some study participants regarded presumptive treatment as poor quality care and preferred diagnostic tests. A young mother said:

“The health worker just looks at the baby and prescribes treatment. How does he know what he is suffering from without tests?”

Some health workers were also alleged to engage in unethical practices including running private businesses within public health facilities and allegations of sale of facility drugs. A young man said:

“…the health worker shows you two types of drugs…. He would say, ‘This drug belongs to the government, and these other one is mine. Mine is the best because it is stronger; so you choose which one you want.’ His drugs are indeed better but more expensive.”

### Advertisements from private drug firms

A range of anti-malarials and other drugs sold over the counter are often the subject of sustained marketing advertisement through the print and electronic media. The continuous advertisement of such drugs by respective companies overshadowed government advertisement of AL the same media. Consequently, community members could not differentiate between the drugs advertised by the private companies and AL as the recommended first-line drug. Concerns about the role of drug companies in limiting uptake of AL were expressed by health workers:

“There is a lot of information coming through radios and posters regarding different types of malaria drugs and without anyone to differentiate for the ordinary people and show them that Coartem is the best and recommended drug… they get confused by the adverts....”

In a few but important cases, the language used in the advertisements by drug companies lacked clarity; for example, among the study community and the wider Coastal region, the term ‘*homa’* can be translated to mean fever, common cold and flu, but it also refers to febrile conditions such as malaria. It was not always clear from the adverts what type of ‘homa’ the advertised drugs treated and so patients often used painkillers to treat malaria with the understanding that they were drugs for ‘homa’. A health worker commented:

“In this community, the word ‘homa’ means different illnesses including common cold, stomach upsets and malaria. Unless these are clarified in the advertisement for drugs, the locals will continue using painkillers in treating malaria....”

## Discussion

There was very limited knowledge within the study district about what prompts changes in treatment policy. Lack of such knowledge led to mostly negative interpretations about why there had been changes in first-line treatment for uncomplicated malaria. Specific to the treatment policy change from SP to AL, the communication strategy employed to facilitate take up of AL had little impact in the study district and instead seemed to perpetuate negative perceptions about the change from SP to AL. Although this study was conducted in 2009, the issues it raises remain important for consideration in future changes in treatment policies and accompanying communication, particularly in remote settings.

One of the things that changing treatment policy and the accompanying communication have to contend with is the entrenched health seeking and treatment habits of the target population. A number of authors such as [14-16] have observed that treatment-seeking behaviour is difficult to change once people get used to particular drugs. Within the study district, use of familiar interventions such as herbs, SP and AQ were widespread and entrenched in people’s treatment culture. It was thus difficult for malaria patients to use AL as they demanded to be given drugs familiar to them. Past studies in Kenya by [7,15] noted that patients demanded for SP instead of AL at health facilities, suggesting poor uptake of AL in the initial stages.

To a large extent, our findings indicate that weaknesses in policy communication during the change from SP to AL contributed to the poor uptake. Part of the reason for the communication problems was that a number of contextual factors relevant to the study population were apparently not considered in the strategy applied. Contextual factors, according to [2] influence health care utilisation, service satisfaction, treatment adherence and health outcomes at all levels. The context include healthcare process (information and its dissemination strategies, health education campaigns and interpersonal engagements) and ethno-social realities (linguistics, health beliefs, socioeconomic status and literacy) [2]. Our findings highlighted similar contextual factors including resource control within households, literacy and health beliefs as well as information packaging and dissemination, and interpersonal communication. Some studies such as [14,16,17] have emphasized the critical role of contextual factors for successful health policy communication. These factors determine which policy communication tools or channels would be most appropriate for a particular setting and require exploration of the target population’s preferred channels of health communication [16].

The government’s overreliance on the print (written) and electronic media meant that communication about the change to AL remained largely inaccessible to most of the study population because of illiteracy and lack of control of such resources as radio and the financial means to maintain them. According to [2], poor communities are least likely to benefit from written health communication because of low levels of education and literacy. In addition, and noting the importance in use of language during health communication, [2] emphasize that provision of health information should go beyond the target group’s preferred language to ensure the information provided matches literacy skills and delivered in a culturally appropriate language. Appropriate use of language could control non-adherence and use of ineffective drugs, which are key catalysts in the development of drug resistance with far-reaching implications for health systems. As noted by [18], practices that lead to development of drug resistance are most common in poor rural areas and so effective communication on drug changes is imperative in such areas to support correct use of drugs.

Recommendations by [2,19] also confirm that effective health policy communication requires multiple channels over a sustained period of time to be able to educate and raise public awareness. Although the change from SP to AL involved multiple channels, it did not include the channel most preferred by the study population, i.e. interpersonal communication involving the local authority, teachers and health workers (particularly public health officers). An important aspect of health policy communication was therefore, not considered during the change from SP to AL: that of engaging the community in the change process. The role and active involvement of the community in health interventions has been emphasized by [5,20,21]. Importantly, delivery of health messages through interpersonal means could have helped in passing consistent information and limited divergent views about AL in the study district. Views that AL was ‘weak’ , ‘strong’ or ‘harmful’ , that it was an experimental drug or from a company after profits, were contradictory and portrayed unintended effects of a health intervention. According to [3], unintended effects following a health intervention can be due to differences in exposure to an intervention as well as in interpretation of information. For the study district, exposure to written and electronic means of communication promoted these unintended consequences because they were largely inaccessible.

Central to interpersonal communication during health policy communication is patient-provider engagement. Community members understood health workers as experts in their field, which explained their strong preference for public health officers as their preferred main sources of health information. Health information communicated by experts, as perceived by the community, is better received compared to communication from ordinary persons [16]. Furthermore, communication between patients and health workers influences treatment outcomes and affects patients’ perceptions of provider competence [22-24]. However, the fact that some patients in possession of AL in the study district referred to it as a painkiller may show the limited information received by patients from providers about their diagnosis and treatment as well as about the presence of new drugs for malaria. For better health outcomes the need for clear, adequate and accurate dialogue between culturally competent providers and patients has been emphasized [2].

Finally, negative perceptions about change of treatment policy for uncomplicated malaria and about AL in particular, could also have emanated from lack of trust in overall political governance given wider media and other reports that described massive high level corruption, for example [25]. Being an economically marginalized district, there seemed to have been little confidence in public initiatives. This may explain why an overwhelming majority of the study participants including a few health workers saw the change in first-line treatment policy from SP to AL as no more than a commercial endeavour.

## Conclusion

The study highlights the need for effective policy communication using locally available channels during the change of first-line treatment for uncomplicated malaria, and perhaps for other drug regimen changes. In addition, communication of the treatment policy change should be supported by other actions such as training health workers to open dialogue and effectively communicate with patients to inspire confidence in new drugs as well as a prolonged public education campaign that allows the public to adjust to the newly introduced drug. Such an approach should be able to increase the amount and quality of information received by beneficiaries, which is important for take-up of new health interventions. Although this study was undertaken only in one district, its detailed investigation of experience suggests that prompt access to effective treatment may not be achieved in any other setting if beneficiaries are not adequately prepared for changes in drug regimens such as first-line treatment for uncomplicated malaria. The study suggests that future changes in first-line drugs should ensure that treatment policy communication is well designed to inspire trust and confidence in the public, particularly among people in remote settings, and to facilitate acceptance and take-up of new health interventions. The choice of a communication channel is as important as the intervention itself.

## Competing interests

The authors declare that they have no competing interests.

## Authors’ contributions

VO designed the study, collected and analysed data and wrote the first and final drafts. LG supported the study design and supervised the entire study. Both authors read and commented on all drafts.
